# The Promising Role of New Generation HDACis in Anti-Cancer Therapies

**DOI:** 10.1016/j.ebiom.2018.05.014

**Published:** 2018-05-11

**Authors:** Eric Hervouet

**Affiliations:** Univ. Bourgogne Franche-Comté, INSERM, EFS BFC, UMR1098, Interactions Hôte-Greffon-Tumeur/Ingénierie Cellulaire et Génique, F-25000 Besançon, France

HDAC inhibitors (HDACis) are small molecules targeting histones deacetylases (HDACs) to lead to increased protein acetylation. To date, 18 HDACs have been identified and classified in 4 different categories based on their degree of homology with yeast counterparts and biochemical properties: Class I (HDAC1, 2, 3 and 8), Class IIa (HDAC4, 5, 7 and 9), Class IIb (HDAC6 and 10), Class III (SIRT1-7) and Class IV (HDAC11). Classes I, II and IV present an activity dependent of Zn^2+^ whereas Class III is NAD^+^ dependent. Since HDAC (HDAC1, HDAC2) expression and activity are frequently increased in cancers and that epigenetics has been shown to be crucial in cancer biology, the project to use HDACis to fight cancer progression emerged more than 15 years ago.

Numerous HDACis have been developed and subdivided in regard of their chemical structure: hydroxamic acids, benzamides, cyclic peptides and fatty acid chains [1]. These molecules are more or less specific to one/several HDACs or one or different HDAC classes. Today, however, only 4 molecules have been approved by the FDA for anti-cancer therapies: i) Vorinostat (SAHA) and ii) Romidepsin (FK288) for cutaneous T-cell lymphoma (CTCL) in 2006 and 2009, respectively, iii) Panobinostat (LBH589) for peripheral TCL (PTCL) and multiple myeloma in 2014 and 2015 and, iv) Belinostat for PTCL in 2014 [2]. The main reasons behind such a low number of HDACis used in anti-cancer therapy are their high toxicity and low specificity. A great deal of effort is currently ongoing to identify better and more specific candidats and more than 350 clinical trials (closed or recruiting, https://clinicaltrials.gov/) are currently assessing the effects of HDACis, alone or in combination with formerly used drugs, in numerous pathologies, especially in cancer. The main goals of these trials are: i) to test new combinations of described anti-cancer drugs (antiproliferative molecules, inhibitors of kinases, DNMTis, HMTis, HDMis…) with HDACis and their effect on cancer progression and, ii) to identify new HDACis showing less toxicity and increased specificity.

The identification and characterization of WW437, a novel hydroxamic acid-derived HDACi, by Zhang and collaborators and published in this issue illustrates the great potential of new generation HDACis. Interestingly, this compound showed no toxicity towards non tumoral MCF10A breast cancer cells but strongly reduced the viability of breast cancer cell lines, with an IC_50_ 10-fold lower than the one observed with SAHA. The strong anti-cancer properties of WW437 were clearly demonstrated since it significantly reduced hallmarks of cancer phenotypes such as proliferation, resistance to apoptosis, migration, invasion, epithelial to mesenchymal transition and tumorigenesis. Moreover, WW437 presented a higher efficiency compared to SAHA in animal models. But even if mice models are largely used worldwide by scientists to evaluate anti-cancer drug properties *in vivo*, these hosts are usually not immunocompetent and effects of the immune response on cancer progression and cross-reactions with the HDACi tested could not be addressed. Indeed, several recent studies reported that HDACis might enhance expression of class I/II MHC complexes and/or favour T-cell mediated killing, suggesting strong interactions between HDACis and cancer immunotherapies [3]. A future evaluation of the potential effects of WW437 in more physiological models such as syngenic or genetic models would help to confirm and definitely validate its *in vivo* anti-cancers properties.

A better understanding of the molecular signaling of HDACis would be required and help in the design of more efficient anti-cancer strategies. Indeed, by inhibiting HDACs, HDACis restore global and/or specific histone acetylation leading to the activation of a subsequent panel of genes by modifying chromatin structure and DNA accessibilitiy to transcriptional factors. Additionnaly, non histone proteins could also be controled by acetylation. For example, the new HDAC3 inhibitor (I-7ab) promotes hyperacetylation of P53 and its transcriptional activity which leads to reduced triple negative breast cancer cell (TNBC) proliferation [4]. In this issue of *EBioMedicine*, Zhang and collaborators elegantly reported that WW437 induced SP1 acetylation by disrupting the interaction of SP1 with HDAC2/4 [5]. As a consequence, SP1 recruitment on the *EphA2* promotor was diminished leading to decreased levels of EphA2, a protein which strongly correlates with agressiveness in breast cancer. The future of this work will require the identification of defined signaling pathways specifically regulated by HDCAis allowing clinicians to design new protocols for future clinical trials. Indeed, future studies should focus on new combinations of newly designed HDCAis, such as WW437, with specific inhibitors of other pathways to induce synergic responses and therefore decrease drug doses. Alternatively, the restoration of gene expression following treatment with HDCAis could also direcly provide new anti-cancer targets. Indeed, this has been previously illustrated by the use of combination of HDACis together with DNMTis which restored response to endocrine therapy in TNBC models [6]. In conclusion, no doubt remains on the potential of new generation HDACis in future anti-cancer therapies but these new anti-cancer therapies will still require long-term strategies and the development of *in silico* modelization to modify already decent HDACis (e.g. WW437) in order to further improve their specificity and efficiency towards cancer cells.

## Disclosure

The author declared no conflicts of interest.
